# Language barriers and cultural differences in childbirth: A qualitative study of Somali women's experiences in Norway

**DOI:** 10.18332/ejm/207799

**Published:** 2025-08-22

**Authors:** Hanna Oommen, Kowsar M. Osman, Miski A. Abdullahi, Mirjam Lukasse

**Affiliations:** 1 Center for Women's, Family and Child Health, Faculty of Health and Social Sciences, University of South-Eastern Norway, Kongsberg, Norway; 2 Department of Research, Sorlandet Hospital Kristiansand, Kristiansand, Norway; 3 Department of Obstetrics and Gynecology, Sorlandet Hospital Kristiansand, Kristiansand, Norway; 4 Department of Nursing and Health Promotion, Faculty of Health Sciences, Oslo Metropolitan University, Oslo, Norway

**Keywords:** experiences, childbirth, language barriers, cultural barriers, Somali women

## Abstract

**INTRODUCTION:**

Somali women represent a significant proportion of the non-Western migrant population in Europe. Language barriers and cultural differences can hinder these women from having a positive childbirth experience. This study aims to explore how Somali women with limited language proficiency experience childbirth in Norway.

**METHODS:**

Social media and snowball method was used to recruit eight Somali women with limited Norwegian language skills who had given birth in Norway within the past three years. Semi-structured, face-to-face interviews were performed during December 2023–January 2024. Thematic analysis according to Braun and Clarke was used.

**RESULTS:**

The study identified three themes: communication and language challenges, relationships with midwives, and cultural differences and health literacy. Communication difficulties, mainly due to language barriers, contributed to feelings of insecurity among the women. Non-verbal communication and husbands acting as interpreters were important for understanding, especially since there were insufficient professional interpreters available. While many women mentioned feeling safe and supported by midwives, some felt alienated and faced stereotypical attitudes. Religious beliefs significantly shaped birthing experiences, leading to a preference for female doctors for both religious and cultural reasons. Limited health literacy contributed to misunderstandings regarding perineal tears and their severity.

**CONCLUSIONS:**

Culturally sensitive care and personalized communication are crucial in preventing misunderstandings and ensuring that vital information is clearly communicated. By enhancing midwives' cultural awareness and multicultural competence, they can offer more tailored and effective support, fostering a positive childbirth experience for women from diverse backgrounds. This approach ultimately leads to improved outcomes and strengthens trust between women and healthcare professionals.

## INTRODUCTION

Over 1 million Somalis have been forcibly displaced, primarily as refugees or asylum seekers, due to ongoing conflict, political instability, and the severe impacts of climate change^1^. Today, Somalis constitute some of the largest migrant communities in Northern European countries^1,2^.

Research indicates that Somali women are at a significantly higher risk of experiencing adverse health outcomes compared to host populations, including stillbirths, prolonged labors often ending in cesarean sections, overdue births, discolored amniotic fluid, and lower use of epidural anaesthesia^3-7^. A critical factor contributing to adverse health outcomes is the high prevalence of female genital mutilation (FGM) among Somali women^8^. Insufficient communication between healthcare providers and Somali women further worsens these risks^9^. In addition, miscommunication due to language barriers can negatively impact patient satisfaction, healthcare quality, and patient safety^10^. Furthermore, research indicates that adequate pain relief and support from healthcare providers, effective communication, and active participation in decision-making, are key factors influencing maternal satisfaction^11,12^.

Clear communication not only conveys critical information but also ensures that a woman feels seen and heard, fostering a positive birth experience^13^. Communication challenges often arise when healthcare providers lack cultural understanding, empathy, or effective communication skills^14^. The WHO framework for improving the quality of care for mothers and newborns identifies eight domains of quality, including two relevant to cultural understanding: effective communication, and respect and preservation of dignity^15^. Effective communication involves delivering information in a culturally sensitive and understandable way, enabling women and their families to make informed decisions, while respect and preservation of dignity entails providing care that is sensitivity to cultural and religious beliefs^15^. When summarizing the findings of women’s views on quality of care, Renfrew et al.^16^ report that women wanted health professionals who combined clinical knowledge and skills with interpersonal and cultural competence. According to the theory of Wikberg^17^, intercultural caring, midwives’ cultural competence, safety, and awareness of bias can lead to tailored care that improves outcomes by adapting to the mother’s needs and the cultural context of care. Midwives’ positive attitude toward Somali migrants can in turn lead to positive and meaningful experiences^18^.

Research shows it is difficult to include Somali women in studies^19,20^. This may partly be due to low Norwegian language proficiency, while limited social contact and unemployment contribute to integration challenges^21^. One Norwegian study about Somali women’s experiences with their first contact with the labor ward showed that the contact was challenging due to one-way communication, poor health literacy, and fear of interventions or adverse outcomes^4^. Despite being one of the largest non-Western immigrant groups in Norway and facing increased risks during pregnancy and childbirth, there is limited research focusing on Somali women’s birth experiences, especially in relation to language barriers and cultural differences. The aim of this study was to investigate how Somali women with limited Norwegian language skills experienced challenges related to language barriers and cultural differences during childbirth.

## METHODS

We conducted a qualitative thematic study by interviewing eight Somali women between 1 December 2023 and 31 January 2024. Two researchers on the team are members of the Somali community in Norway and have personal experience with cultural barriers. Both are native Somali speakers. This shared background with the participants, along with the opportunity to conduct interviews in Somali, contributed to open communication and a sense of security and trust.

Recruitment began with posts in the Facebook groups ‘Somali Mothers’ (3000 members) and ‘Somalis in Norway’ (7000 members) in December 2023, aiming to reach women within the community. Initial attempts to recruit participants through cultural meeting points, such as mosques and women’s health centers, were unsuccessful, as participants were skeptical. The breakthrough came when our first participant was referred by an acquaintance. Building on this initial connection, we adopted the snowball sampling method^22^, allowing us to leverage existing trust within the community. Participants were eligible for inclusion if they were Somali women aged >18 years who had given birth in Norway within the past three years, but at least two months before the interview. They were included if they self-reported limited Norwegian language skills at the time of birth and identified themselves as Somali. Their level of written and spoken Somali was not considered for inclusion, nor how long they had been living in Norway.

An interview guide for semi-structured interviews was developed with eight open-ended questions. The guide was a flexible tool to ensure relevant topics were covered while allowing for follow-up questions. The interview guide was reviewed and adjusted after the first two interviews. Women tended to give short answers, and prepared follow-up questions had to be used (Supplementary file).

The interviews were conducted in person at locations the participants chose for their comfort. Three interviews took place in the woman’s home, one in the local library, one in a café, and three in a mosque. The two Somali authors were present at all interviews. The person not conducting the interview took notes and assisted the person performing the interview to ensure nothing was forgotten or misunderstood. The interviews lasted on average 39 minutes, the shortest lasting 29 minutes and the longest 50 minutes.

The recorded interviews were transcribed directly from Somali to Norwegian, resulting in 54 pages of transcription. Considering the data collected after 8 interviews, it was determined that sufficient data had been collected to effectively meet the aim of our study. Each Somali author transcribed half of the interviews and checked the transcription of the other to ensure accuracy and capture nuances during translation.

The study was submitted for evaluation by Norway’s governmental body overseeing data collection and privacy (Sikt, Ref.nr.180235) in December 2023. As recalling childbirth can be traumatic, a contingency plan was in place for referrals to professional psychological care if needed. Before the interview, participants received verbal and written information on privacy protections under Norwegian medical research law. They were informed of their right to withdraw before their data was integrated. To protect women’s confidentiality, recordings were de-identified using a key-number system, with background information collected separately. Interviews were recorded via the Diktafon app and securely stored in the University of Oslo’s cloud service (Nettskjema.no). All participants provided written consent, and the interviewer remained attentive to potential emotional distress, offering to pause if necessary.

### Analysis

Based on the six steps of Braun and Clarke^23^, thematic analysis was used to analyze the data. An inductive approach to analysis was employed, allowing themes and subthemes to emerge from the data. The initial stage of analysis involved familiarization with the data through transcription, repeated reading, and reflection. The dataset was systematically examined in the second step, ensuring careful attention to all its elements. Upon solid familiarization with the data, generating initial codes began. The third phase included identifying themes and sorting codes under themes. Fourth, subthemes were identified and organized under overarching main themes. Finally, the main themes and subthemes were labelled. The analysis process was iterative, with themes and subthemes continuously being refined to ensure clarity and coherence. Overlaps were minimized, and labels were adjusted until the themes and subthemes accurately and systematically represented the findings while remaining faithful to the participants’ accounts.

## RESULTS

The final sample included eight multiparous women, aged 29–44 years, who had lived in Norway for 6–17 years and completed primary school or lower (Table 1). Two of the women did not have a support person present at the birth. Four of the eight women had completed 8 years of primary school. Three main themes were identified, each with three subthemes as shown in Table 2.

**Table 1:** Characteristics of the eight participants in the qualitative study on Somali women’s experiences with maternity care in Norway, December 2023 – January 2024

*Participant*	*Number of children*	*Mother’s birth year*	*Year came to Norway*	*Companion during childbirth*	*Education level*	*The year of birth of the last child*
1	6	1984	2009	Husband	Not completed primary school	2023
2	3	1995	2016	Husband	Not completed primary school	2022
3	2	1992	2011	Husband	Primary school	2021
4	2	1985	2008	Friend	Primary school	2021
5	3	1993	2018	Husband	Primary school	2022
6	4	1984	2014	None	Not completed primary school	2022
7	3	1985	2018	Husband	Not completed primary school	2021
8	4	1980	2007	None	Primary school	2022

**Table 2:** Final themes and subthemes in the qualitative study on Somali women’s experiences with maternity care in Norway, December 2023 – January 2024

*Themes*	*Communication and language challenges*	*Relationships with midwives*	*Cultural differences and health literacy*
**Subthemes**	Communication challenges Preference for health professionals who speak Somali Different methods and tools for translating	Care, attention, and security Disrespect and prejudices Husband as protection	Somali traditions Comparison between Norway and Somalia Health literacy

### Communication and language challenges

Limited Norwegian proficiency at the time of birth was an inclusion criterion, with participants self-assessing their language skills. While some initially said understanding was not an issue, as the interviews progressed, they recalled challenges with specific terms, particularly birth-related ones like ‘contractions’. As one participant noted:

*‘The word contractions was new to me. That’s how I learned it.’* (Participant 3)

Women found it both frustrating and problematic not being able to express themselves fully. They mentioned avoiding responding to questions out of fear of saying something wrong or just answering ‘*everything is ok*’ to stop a conversation they did not feel comfortable with. Women explained that despite understanding what the midwife asked, they did not dare to answer for fear of not being able to express themselves and that this could lead to medical errors. As one woman said:

*‘If you do not trust yourself and think, what if I say something wrong so that I will be injected with the wrong medicine. You have a lot of thoughts, and you become afraid.’* (Participant 8)

The women reported that healthcare staff made significant efforts to communicate effectively. Women agreed that it was easier to communicate face-to-face even though they did not speak the language as non-verbal communication was used. Midwives demonstrated and used equipment to support communication. Women pointed out that the midwives used physical touch to reassure them. This made women feel understood and cared for:

*‘She came to me, stroked my arm, calmed me down, saying, “It’s going to be okay; we’ll help you”.’* (Participant 8)

However, two participants mentioned instances where they lacked sufficient information during childbirth. Both women’s childbirths had been attended by a student midwife without prior clarification, leaving them wishing for the expertise of a qualified midwife.

Participants expressed a strong preference for Somali-speaking midwives for two reasons. Firstly, they would have been able to express themselves freely and understand the information given. Secondly, this improved communication would have had the potential to prevent unwanted outcomes during birth and in the postnatal period. One woman whose birth was complicated by an anal sphincter injury wondered if it could have been prevented if she and the midwife had spoken the same language. A couple of women remained uncertain about fully understanding the Norwegian-speaking midwives, even if they grasped most of the information. As one woman stated:

*‘When you speak the same language, there are many things you can ask a person.’* (Participant 1)

Most of the participants relied on their husbands as interpreters and trusted their translations entirely:

*‘I had full trust in him.’* (Participant 4)

Those who were accompanied by their husband expressed no need for an interpreter. They reported that it did not matter that the midwife talked to their husband instead of directly to them. As one woman said:

*‘If you have a 100% trust in the person, it is the same for me, if it is him or me who is talking. To understand is the most important.’* (Participant 5)

Women relied heavily on their husbands to repeat the information given by the midwife. Being in labor made it difficult to concentrate on what the midwife said. However, the absence of husbands due to COVID-19 restrictions posed a significant challenge for some women, leading to anxiety about communication:

*‘I thought to myself, how will I manage to speak?’* (Participant 2)

In the absence of their husbands, women reported relying on alternative resources for communication, including Somali-speaking staff, Google Translate and, in one case, written information in Somali.

### The relationships with midwives

A common theme for all the women was the importance of a good relationship with their midwife. This was particularly crucial when they did not have a support person. As one woman shared:

*‘When the midwife is with me, the pain feels less … but when they leave, I start feeling the pain again.’* (Participant 8)

Most women felt well-cared for despite the language barrier, with midwives providing encouragement, necessary information, and addressing all their needs. They expressed gratitude for the care they received. Women told about being warmly welcomed and met with openness. Even though women could not speak the language they reported receiving encouragement from the midwives throughout labor. Midwives were described as providing information, food, and drink, and performing other midwifery tasks with compassion.

However, some women reported negative experiences, feeling alienated and treated in a degrading way. The women who mentioned this, experienced their midwife as discriminating and judgmental, stereotyping them. Women sensed the midwives’ negative attitude towards them for having many children and wearing traditional clothing. One woman avoided making noise during labor, fearing the midwife’s reaction:

*‘You are not respected … it makes you feel like never wanting to give birth again.’* (Participant 8)

Others had mixed experiences, depending on the midwife on duty. Some midwives were kind and attentive, while others seemed uncaring and lacked compassion, which the women linked to language barriers and their ethnicity.

Most women viewed their husbands as a source of protection and support, especially because of their husband’s ability to communicate in Norwegian. They appreciated their husband being present throughout. During COVID restrictions, two women struggled without their husbands present, and felt unsafe due to their limited Norwegian. They argued with the staff to allow the husband into the labor ward. After birth, the husband had to leave again and could only come in during visiting hours. One woman felt badly treated by the midwife and was convinced that her husband would have prevented this:

*‘Since she saw my poor language, that I cannot say more than two words joined together. She would not have pushed me away on that ground I mean. My husband would have stopped her.’* (Participant 2)

### Cultural differences and health literacy

Women explained that in Somalia the family comes with food. The hospital food was different from what the women were used to at home, with several describing it as mostly bread, boiled potatoes, and squash (diluted artificial fruit drink). As a result, many received warm food from home when possible. Even though women felt there was little variation in the food they could chose, they appreciated that it was free of charge. The choice of food became additional limited as it had to be halal (Arabic for lawful or permitted under Islamic law). One woman said:

*‘The food, eh … I did not receive special my own food. There were times when the food wasn’t halal. Everybody can eat bread and find halal stuff to put on it. But sometimes when you count on dinner and go to eat and find that you cannot eat it because it is not halal. Then you do not have dinner.’* (Participant 8)

Norway has few male midwives, so the gender of midwives was not an issue. Most women found having a male doctor acceptable during delivery, as they considered their care as life-saving during labor. The health of the mother and baby was most important. However, a female doctor was preferred for cultural and religious reasons. One woman expressed her discomfort:

*‘Wallahi (I swear by Allah), yes. When they asked me to spread my legs, it was very difficult for me. I had not done this before. I saw it as shameful. I was embarrassed, Wallahi.’* (Participant 3)

When comparing Norway to Somalia, women preferred giving birth in Norway. Women felt that it was safer to give birth in Norway. They explained that Norway is a developed country with good education. They said that in Somalia childbirth is associated with many complications. They told stories about women giving birth at home with an uneducated midwife or older woman bringing along just a scalpel for the birth. This person cannot give any pain relief nor stop any bleeding. In addition, these women use thick suture material which does not dissolve by itself. One woman described the difference:

*‘Here, you receive a lot of care and help, and you are looked after well and your baby. But in that country, it is each person for herself, and it depends on your wealth. There is no one to care for you. In contrast, here, the government is responsible for the people, and doctors work hard to help you.’* (Participant 6)

Women mentioned the closer involvement of the family in childbirth compared to Norway. Several family members can be present at the birth. They described how midwives felt like relatives as they had a similar background. While some appreciated the presence of family members during childbirth in their home country, one woman appreciated giving birth in Norway, where only her husband and the midwife were present, avoiding feelings of shame from too many people.

Women seemed to lack knowledge about their body in relation to childbirth. Several women experienced perineal tearing during childbirth, and their understanding of the issue varied. One woman expressed confusion:

*‘They stitched me up well, but I don't understand why I get tears every time I give birth.’* (Participant 6)

Another woman misunderstood the degree of tearing, saying:

*‘She stitched me for about 7 or 2 meters.’* (Participant 2)

highlighting a lack of understanding of the metric system. Another woman, dissatisfied with the lack of support of her perineum, mentioned receiving four stitches and later physiotherapy. When asked to clarify, her description confirmed she had a grade 4 perineal tear (Participant 1).

## DISCUSSION

This study identified three main themes that had an impact on Somali women’s childbirth experiences in Norway: 1) communication and language challenges; 2) the relationships with midwives; and 3) cultural differences and health literacy.

The majority of the Somali women in our study had lived in Norway for more than 5 years, four of them more than 10 years, before the birth they were interviewed, yet they continued to face communication challenges that affected their birth experiences. Our findings align with previous studies that have identified language barriers as a primary cause of miscommunication between patients and healthcare providers during pregnancy and childbirth^4,10,14,18^. Such barriers can negatively impact the quality of care and patient satisfaction^4,10,14,18^. Research further indicates that healthcare providers may overestimate migrant patients’ language proficiency, leading to reduced use of professional interpreters and, consequently, communication difficulties^24^. In our study, due to the women’s long-term residency in Norway, healthcare providers may have assumed they were proficient in the Norwegian language. However, research has found that even when language issues are obvious there is insufficient use of interpreter services^24^.

While the majority of women in our study said they understood the information given during childbirth, uncertainty arose due to unfamiliar medical terms like ‘contractions or perineal tears’. This aligns with the King-Shier et al.^25^ study, where patients reported difficulty understanding medical terminology, even with adequate language skills. Effective communication techniques, such as speaking slowly and using simple language, and use of digital tools may improve patient comprehension^10^. Additionally, non-verbal communication was highlighted by several participants as helpful, with gestures and eye contact providing reassurance and understanding. Similar findings were reported by Carroll et al.^26^, where Somali women found non-verbal cues critical for feeling heard and supported.

The women in our study also underscored the critical role their husbands played as emotional and communication support. This aligns with the review of Bohren et al.^27^, which highlights the significance of continuous companionship during labor. Companions, such as partners or chosen support persons, can help bridge communication gaps, provide non-pharmacological pain relief, and share crucial information about labor processes^27^. However, using a husband as an interpreter poses risks, as unqualified interpreters may withhold or misinterpret critical information^28^. The linguistic concerns and lack of adequate provision of language support among migrant women have been discussed in other studies^18^. Tailored information, language support, flexible routines and alternative support mechanisms, such as multicultural doula intervention, which have shown promising results, may help to improve perinatal outcomes and experiences for this group^29,30^. It is important to note that communication challenges can exacerbate other aspects of the childbirth experience. For instance, language barriers may affect pain management, as highlighted by previous research^31^. Simultaneously, intense pain can further impede effective communication, especially for individuals already facing linguistic difficulties^7,32^. These interconnected factors emphasize the need for targeted interventions that address both communication support and labor companionship for migrant women.

Our participants also shared mixed experiences regarding the involvement of midwifery students during childbirth. While there is limited evidence specifically advising against student involvement in migrant women’s care, studies underscore the importance of culturally competent care for this population^33^. This raises important questions about the preparedness of midwifery students to address the complex needs of migrant women. Comprehensive cultural competence training for all healthcare providers, including students, is essential. It may also be worth considering whether students doing their final practice should participate in the care of migrant women, given the unique challenges faced by this group.

Language and cultural barriers emerged as significant factors influencing not only communication between women and midwives but also the quality of their relationships. Our study showed that these barriers affected the sense of safety and trust during childbirth, which aligns with findings from previous studies^34^. Our study reinforces that respectful and culturally sensitive care by midwives fosters trust and a sense of safety, leading to more positive birth experiences for women. These results align closely with the framework for quality maternal and newborn care, which emphasizes evidence-based, person-centered care to promote equity, safety, and respectful maternity care^16^. Conversely, some participants in our study reported encountering midwives with prejudices, which negatively impacted their birth experiences. Discriminatory comments about ethnicity or family size fostered feelings of alienation and exclusion, consistent with a study from UK that highlighted how such behavior worsens vulnerability and weakens trust during childbirth^18^. A noteworthy finding was the husband’s expanded role, often serving not only as an emotional support figure but also as a protector and language bridge between women and midwives, an interaction also described in other maternal care research^35^.

When women compared healthcare in Somalia and Norway, they perceived childbirth as safer in Norway. Still, they noted that, while Somali women often prefer female doctors, this preference is not always accommodated in acute situations. In contrast, Somali traditions placed greater emphasis on family involvement during the childbirth process, highlighting cultural differences between the two countries. These findings align with other studies comparing Western and Somali childbirth practices^36^. To ensure Somali women have a positive birth experience, increasing cultural awareness among healthcare providers, alongside linguistic assistance and respectful care, emerges as a priority.

### Strengths and limitations

A strength of this study is that it collects firsthand accounts from Somali women in their native language, addressing a gap in previous research. Conducting the study in Somali allows participants to share their experiences more authentically, fostering trust and providing more accurate insights into the challenges they face during childbirth. This approach enhances the depth and accuracy of the data, providing valuable insights into the challenges faced by Somali women during childbirth. Additionally, having Somali-speaking researchers involved in the transcription and translation process helps preserve the nuances of participants’ narratives, reducing the risk of misinterpretation. The help of a ‘gate-keeper’ at the mosque was essential in recruitment.

However, the study also has certain limitations. The study is small with only 8 participants. While we considered that we had sufficient data to investigate our aim, we are uncertain if saturation was reached. One challenge was that participants were not accustomed to providing detailed descriptions of their experiences. Even when interviewed in their native language, many gave brief responses without elaboration, which may have limited the depth of the findings. Neither did we perform a member check to ensure correct data. Another limitation is the study’s transferability, as the findings may not be generalizable beyond Somali women with similar migration backgrounds and experiences in Norway. Additionally, cultural and social norms may have influenced participants’ willingness to discuss certain aspects of their childbirth experiences openly.

## CONCLUSIONS

This study highlights the critical role of addressing language barriers, cultural competence, and health literacy to improve the birthing experiences of Somali women. Ensuring access to professional interpreters, fostering culturally sensitive care, and bridging gaps in health education are essential steps to create a supportive and inclusive maternity care environment. Flexible attractive language learning opportunities for women with children with limited possibilities to attend classes at locations some distance from home could be developed and tested. Future research could explore the impact of newly developed health information available in Somali in Norway, as well as programs designed to support migrant women.

## Supplementary Material



## Data Availability

The data supporting this research cannot be made available for privacy reasons.
