# Supplementary material for: Language barriers and cultural differences in childbirth: A qualitative study of Somali women's experiences in Norway
Source: Eur J Midwifery. 2025 Aug 22;9:10.18332/ejm/207799. doi: 10.18332/ejm/207799 (PMC12371482; doi:10.18332/ejm/207799)
Supplement: Supplementary file 1 [file EJM-9-37-s1.pdf]

Supplementary file 1

Interview guide

Birth experience

1. What thoughts did you have about childbirth in Norway before you gave birth here (practice and culture)?
2. Can you tell us about your experience of the birth (on arrival, during labour and immediately after birth)?
3. How was the contact with healthcare professionals during childbirth? Communication, language - did you understand the information provided? Were there any misunderstandings? Feel free to elaborate.
4. Were you informed about the measures and examinations that were carried out? How did they do it?
   - If yes, how was this explained to you?
   - If no, did you ask what the healthcare professional was doing?
5. Did you have someone with you in labour? Family, friends (why did you choose to bring this person, whose friends or others?) (do they speak Norwegian/English), interpreter, doula
6. How did you feel you were met? Were you well looked after? Safety, cultural respect.
   - Can you give examples of things that healthcare professionals did that made you feel cared for and safe?
7. Did you feel unsafe and afraid at any point during the birth - What was the triggering factor and how was this dealt with by the midwife or whoever was with you in labour?
8. Do you think the birth would have been different if you and the midwife had spoken the same language - is there anything you kept to yourself in labour because you didn't speak the language well enough?
9. Has anything happened or have you experienced anything that is not common in your culture during this birth? If yes, what happened and how did you react to it? - The food at the hospital. Male health professionals.
10. Is there anything else you would like to share with us related to the birth?

© 2025 Oommen H. et al.
